# A Mucinous Cystic Neoplasm of the Mesocolon Showing Features of Malignancy

**DOI:** 10.1155/2012/727105

**Published:** 2012-10-18

**Authors:** Kiki Mistry, Marta Penna, Shiva Dindyal, Hasan Mukhtar

**Affiliations:** Department of Surgery, Whittington Hospital, London N19 5NF, UK

## Abstract

Mucinous cystic neoplasms are rare tumours of uncertain histogenesis. They arise from the ovaries, pancreas, and other intra-abdominal sites but more unusually from the mesocolon. They can present with abdominal pain, distension, or a palpable mass but are commonly an incidental finding. We describe the case of a 48-year-old woman who was found to have an incidental left pelvic cyst on computed tomography. Subsequent laparoscopic excision and histological analysis demonstrated the cyst to be a borderline malignant mucinous tumour arising from the mesocolon. Mucinous tumours should be considered in the differential diagnosis of all intra-abdominal cysts and treatment should be by surgical complete excision.

## 1. Introduction

Mucinous cystic neoplasms (MCNs) can arise from the ovary and other extraovarian sites including the pancreas, liver, kidneys, and appendix but more rarely from the mesentery. These mesenteric neoplasms are classified into benign cystadenomas, borderline tumours, and invasive carcinomas according to the presence of malignant features on histology. There are very few reported cases of mucinous cystic neoplasms of the mesocolon in the literature; moreover, those with features of malignancy are rarer still.

## 2. Case

**Figure 1:**
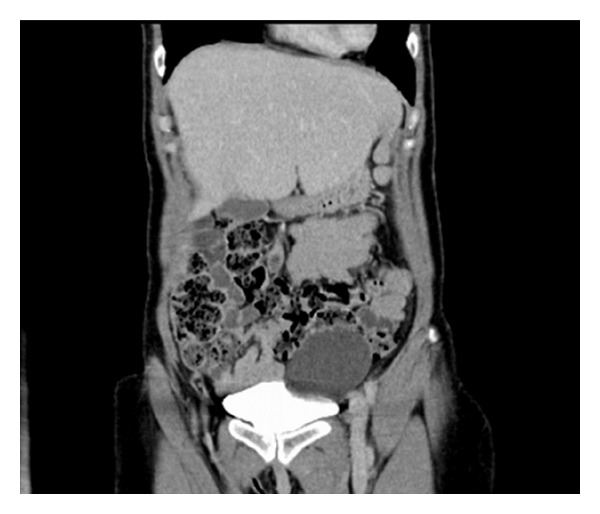
Coronal view of cyst showing its relationship with the sigmoid colon on computed tomography.

A 48-year-old woman presented to our urologists with haematuria secondary to a renal calculus. Computed tomography (CT) revealed a 5 mm ureteric calculus and an incidental 71 mm by 57 mm cyst in the left pelvis that was initially believed to be an ovarian cyst (Figure 1). 

She was asymptomatic with respect to the cyst and had no relevant medical problems. There was a strong family history of pancreatic cancer on her paternal side and her father died of pancreatic cancer aged 57. There was minimal tenderness on deep palpation of her left iliac fossa. Routine blood tests were normal, and tumour markers CA19.9 and CEA were within normal limits.

Transvaginal ultrasonography revealed a left adnexal cystic mass with slight thickening of the walls and anterior wall trabeculae. It was unclear whether this cystic mass was arising from the ovary itself. Magnetic resonance imaging of the abdomen was organised to define the anatomical relationship between these structures, which suggested a large simple cyst, possibly left paraovarian. It had increased in size to 82 mm × 65 mm × 105 mm since the initial CT scan six months earlier.

At laparoscopy, the cyst was attached to the sigmoid mesocolon. The cyst was enucleated and 700 mls of fluid aspirated for cytology. The sigmoid colon and its mesentery were viable; thus, resection was avoided.

Macroscopic examination showed a grey thin-walled, unilocular cyst, which was microscopically composed of dense fibroconnective tissue and ovarian-type stroma. The cyst was lined by mucin-secreting epithelium of intestinal type, including goblet cells. In the majority of areas, there was variable cellular stratification with formation of fused, cribriform structures and arborizing papillary formations. There were varying degrees of cytological atypia associated with increased mitotic activity. On immunohistochemical staining, there was widespread positivity to both cytokeratin-7 and -20.

She was discharged home after an uneventful postoperative period. Her histology was discussed at our multidisciplinary meeting where it was recommended that she have a follow-up CT specifically focusing on her pancreas given her positive family history. 

## 3. Discussion

Mucinous cystic neoplasms of the mesocolon are rare intra-abdominal lesions in the spectrum of mesenteric cysts [1]. The majority of cases of MCN have been described in women [2]. They are commonly detected incidentally but can present with chronic abdominal pain, distension, or an abdominal mass. These tumours pose a diagnostic challenge due to their lack of specific symptoms, biochemical markers, and radiological features. Despite the abundance of imaging modalities used in our case, it was only at laparoscopy that the cyst was demonstrated to be arising from the sigmoid mesentery. In the majority of cases, the diagnosis of MCN is made postoperatively after histological examination. In a recent review by Cauchy et al., preoperative histological examination of the cyst by fine needle aspiration or biopsy was not recommended due to the risk of peritoneal spillage and tract seeding [3].

MCNs are histologically similar to ovarian mucinous cystadenomas [4]. The cyst is composed of an outer wall of ovarian-like stroma consisting of spindle-shaped cells and myofibroblastic proliferation and an inner layer of mucin-secreting columnar and cuboidal cells [5]. The identification of ovarian-like stroma on histological examination is diagnostic of mucinous cystadenomas, however, its absence does not preclude the diagnosis. Cysts of borderline malignancy display nuclear atypia and increased mitotic activity. Several hypotheses exist regarding the pathogenesis of MCNs, the most popular being that they develop from invaginations of the peritoneum that form inclusion cysts [6]. The epithelial lining of these cysts subsequently undergoes metaplasia forming MCNs. Other proposals are that given their similarity with ovarian cystadenomas they are formed from ectopic ovarian tissue [7].

The treatment of choice is complete surgical excision given the malignant potential of these cysts [1]. It permits comprehensive histological examination and reduces the risk of recurrence. Excision can be by open or laparoscopic surgery, the latter being favoured by a recent review of mesenteric cysts [8]. 

MCNs are uncommon tumours that are difficult to diagnose preoperatively. Their pathoneogenesis remains unclear. Given their malignant potential, they are an important differential for all intra-abdominal cysts. We recommend that the definitive treatment for all MCNs should be complete excision. This can be performed openly or laparoscopically depending on the size of the cyst, its location, and the skill of the surgeon.
